# Adjunctive dexamethasone implant during vitrectomy with silicone oil for diabetic tractional retinal detachment: a systematic review and exploratory meta-analysis

**DOI:** 10.1186/s40942-026-00903-5

**Published:** 2026-07-21

**Authors:** Ahmar Jan Qureshi, Ali Shan Hafeez, Hamna Hassan Siddiqui, Ahsab Zaka, Muhammad Riyyan Tariq Tagga, Tooba Munir, Eashel Farooq, Muhammad Bilal, Atiqullah Sadaqat

**Affiliations:** 1 CMH Multan Institute of Medical Sciences, Multan, Pakistan; 2 Jinnah Sindh Medical University, Karachi, Pakistan; 3 Services Institute of Medical Sciences, Lahore, Pakistan; 4 Liaquat University of Medical and Health Sciences, Jamshoro, Pakistan; 5 Sargodha Medical College, Sargodha, Pakistan; 6 Department of Curative Medicine, Faculty of Medicine, Kateb University, Kabul, Afghanistan

**Keywords:** Diabetic tractional retinal detachment, Pars plana vitrectomy, Silicone oil tamponade, Dexamethasone implant, Proliferative vitreoretinopathy

## Abstract

**Background:**

Diabetic tractional retinal detachment (TRD) secondary to proliferative diabetic retinopathy is a vision-threatening complication that may require pars plana vitrectomy in selected cases, particularly when the macula is threatened or detached. Silicone oil tamponade is commonly used in complex cases, although tamponade choice varies according to surgical findings and surgeon preference.

**Objectives:**

To evaluate surgical outcomes associated with adjunctive dexamethasone implant during pars plana vitrectomy with silicone oil or heavy silicone oil tamponade for diabetic tractional retinal detachment.

**Methods:**

A comprehensive literature search of PubMed, Scopus, Web of Science, and ClinicalTrials.gov was performed from inception through 2025. Randomized and non-randomized comparative studies assessing PPV with silicone oil with or without adjunctive dexamethasone implant were considered. Definitions of postoperative PVR and retinal redetachment were accepted as reported by the original studies; no individual-level imaging or independent photographic regrading was available. The anatomical outcome was an exploratory composite of study-reported postoperative proliferative vitreoretinopathy (PVR) and/or retinal redetachment outcomes. Because definitions and follow-up time points differed across studies, this outcome was not treated as a uniformly graded PVR endpoint. Randomized and non-randomized comparative studies were identified; randomized trials were prioritized for the main analysis, while the non-randomized study was considered separately because of serious risk of bias. Secondary outcomes included best-corrected visual acuity (BCVA), central subfield thickness (CST), ocular hypertension, and postoperative complications. The protocol was prospectively registered in the International Prospective Register of Systematic Reviews (PROSPERO: CRD420261283105) prior to the commencement of the review.

**Results:**

Four studies involving 305 eyes were included. In the main randomized-trial analysis, two studies comprising 123 eyes contributed to the exploratory study-reported postoperative proliferative/redetachment composite. The pooled estimate was highly imprecise (RR 0.20, 95% CI 0.00 to 16.06; I² = 0%) and did not provide conclusive evidence of benefit. In an exploratory sensitivity analysis including the non-randomized study, the estimate favored dexamethasone implant, but this analysis was subject to serious risk of bias. BCVA and CST findings were limited by small sample sizes, heterogeneity, and inconsistent reporting. Intraocular-pressure outcomes were not pooled because definitions and assessment time points differed across studies.

**Conclusion:**

Current evidence is insufficient to support routine adjunctive dexamethasone implant use during vitrectomy with silicone oil for diabetic tractional retinal detachment. The available studies provide only a hypothesis-generating signal of fewer study-reported postoperative proliferative or redetachment events. Use should remain selective and individualized until adequately powered randomized trials with standardized anatomical outcomes are available.

**Clinical trials number:**

Not applicable.

**Supplementary Information:**

The online version contains supplementary material available at https://doi.org/10.1186/s40942-026-00903-5.

## Introduction

Diabetic tractional retinal detachment (TRD) is a severe vision-threatening complication of proliferative diabetic retinopathy (PDR), characterized by fibrovascular proliferation and contraction that exerts traction on the retina, resulting in macular detachment and profound visual loss [1, 2]. It represents the most advanced stage of diabetic retinopathy and typically occurs in patients with long-standing, poorly controlled diabetes. Globally, diabetic retinopathy affects approximately 22–35% of people with diabetes, while PDR develops in 3–8% of these patients. Among those with PDR, tractional retinal detachment occurs in approximately 5% of cases, making TRD a major cause of irreversible blindness in the working-age population worldwide [3, 4].

Management depends on macular status, extent and progression of traction, presence of combined rhegmatogenous components, vitreous hemorrhage, and surgical risk. Extramacular or stable TRD may be observed, whereas progressive macula‑threatening or macula‑involving TRD is generally considered for PPV. Silicone oil is often selected in complex cases requiring long‑term tamponade, but gas tamponade may be appropriate in selected eyes.

Despite advances in surgical technique, the management of diabetic TRD remains challenging. Anatomical failure after diabetic TRD surgery may result from recurrent traction, residual or recurrent fibrovascular proliferation, iatrogenic or pre‑existing retinal breaks, combined rhegmatogenous detachment, and postoperative PVR [5, 6]. Distinguishing new postoperative PVR from persistent pre‑existing tractional pathology is often difficult in published studies.

In diabetic tractional retinal detachment, postoperative anatomical failure may result from residual traction, recurrent fibrovascular proliferation, iatrogenic retinal breaks, combined traction-rhegmatogenous detachment, or true postoperative PVR. These entities are not interchangeable. Unlike classic PVR after rhegmatogenous retinal detachment, postoperative membrane formation in diabetic tractional retinal detachment may overlap with pre-existing diabetic fibrovascular disease.

Additional complications, including postoperative ocular hypertension, macular edema, and anterior segment inflammation, further compromise visual recovery. These limitations underscore the urgent need for effective adjunctive therapies that can safely reduce the inflammatory and fibrotic response driving PVR.

The intravitreal dexamethasone implant (Ozurdex^®^), a biodegradable sustained-release corticosteroid, offers a promising adjunctive strategy due to its potent anti-inflammatory and anti-fibrotic effects. By suppressing the release of pro-inflammatory cytokines and growth factors, it may mitigate postoperative intraocular inflammation in vitrectomized eyes [6]. Recent prospective randomized controlled trials and comparative studies have evaluated its use at the end of PPV with silicone oil tamponade, reporting potentially favorable postoperative anatomical and inflammatory outcomes; however, the available studies are small and use variable outcome definitions and follow-up periods [2, 4–6]. Individual studies have been relatively small, with variation in reported effect sizes and follow-up duration, resulting in inconsistent conclusions regarding the magnitude of benefit.

This systematic review and exploratory meta‑analysis summarizes the available comparative evidence and estimates the direction and magnitude of association between adjunctive dexamethasone implant and postoperative anatomical, visual, and safety outcomes. Given the small evidence base, the analysis is intended to identify signals of benefit and uncertainty rather than to provide definitive treatment estimates.

## Methods

This systematic review and meta-analysis was conducted in accordance with the Cochrane Handbook for Systematic Reviews of Interventions [7] and reported in accordance with the PRISMA 2020 guidelines [8]. The protocol was prospectively registered in the International Prospective Register of Systematic Reviews (PROSPERO: CRD420261283105) prior to the commencement of the review.

### Search strategy

A comprehensive search of PubMed, Scopus, Web of Science, and ClinicalTrials.gov was conducted independently by two reviewers to identify studies evaluating the effect of adding an intravitreal dexamethasone implant to pars plana vitrectomy (PPV) with silicone oil (SO) or heavy silicone oil (HSO) tamponade in patients with diabetic tractional retinal detachment (TRD). The search was performed from database inception to 29 December 2025, using relevant keywords and controlled vocabulary terms. The search strategy included terms related to diabetic TRD, pars plana vitrectomy, silicone oil tamponade, and dexamethasone implant (Ozurdex). The final search was conducted on 29 December 2025. The core PubMed strategy was: ((“diabetic tractional retinal detachment“[tiab] OR “tractional retinal detachment“[tiab] OR “TRD“[tiab])) AND ((“pars plana vitrectomy“[tiab] OR PPV[tiab]) OR silicone[tiab] OR “silicone oil“[tiab] OR “heavy silicone oil“[tiab]) AND (“dexamethasone implant“[tiab] OR Ozurdex[tiab] OR dexamethasone[tiab]). ClinicalTrials.gov and reference lists of included studies and relevant reviews were searched. Preprint servers were not searched. Grey-literature searching was limited to ClinicalTrials.gov. Studies in languages other than English were eligible if an English abstract was available; full-text translation was not undertaken. The full search strategies for each database are provided in the Supplementary Materials (Supplementary Table 1).

### Study selection criteria

Studies were eligible for inclusion if they met all of the following criteria: (i) enrolled adult participants with diabetic TRD secondary to proliferative diabetic retinopathy, confirmed by fundus examination or optical coherence tomography (OCT); (ii) compared the intervention of PPV + SO/HSO tamponade + intravitreal dexamethasone implant (at the time of surgery or postoperatively) against a comparator of PPV + SO/HSO tamponade alone; (iii) employed a randomized controlled trial (RCT), non-randomized comparative study, prospective cohort, or retrospective cohort design; and (iv) eligible studies were required to report at least one anatomical outcome, preferably retinal redetachment attributable to PVR grade C or worse, need for reoperation due to PVR, or study‑defined postoperative PVR. Grade B PVR alone was not considered a robust primary endpoint because retinal surface wrinkling, stiffness, vessel tortuosity, and membrane‑related distortion may overlap with baseline diabetic tractional pathology. Studies were excluded if they: (i) were case series without a comparator group; (ii) did not involve TRD (e.g., simple rhegmatogenous retinal detachment only); (iii) did not use silicone oil or HSO as tamponade; (iv) involved dexamethasone implant use for macular edema only in the absence of TRD; or (v) consisted of non-primary research such as reviews, editorials, or conference abstracts without original data.

Clinically significant PVR was defined as grade C or worse PVR, retinal redetachment attributed to PVR, or reoperation undertaken for PVR. However, because the included studies used non-equivalent definitions and did not always distinguish PVR from residual traction or recurrent fibrovascular proliferation, the pooled anatomical analysis was treated as exploratory.

### Study selection process

All identified records underwent a two-stage screening process. Titles and abstracts were screened independently by two reviewers (A.Z., M.R.T.T.) using pre-specified eligibility criteria. Full-text assessment of potentially eligible studies was then performed independently by the same reviewers. Any disagreements at either stage were resolved through discussion or by consultation with a third reviewer (A.J.Q.). Reference lists of included studies and relevant systematic reviews were screened to identify additional eligible records. The study selection process is summarized in the PRISMA flow diagram (Supplementary Fig. 1).

### Data extraction

Data were extracted independently by two reviewers (A.J.Q., H.H.) using a standardized, piloted extraction form. Extracted data included: study characteristics (authors, year, country, design, sample size, enrollment period), patient demographics and clinical details (age, sex, diabetes duration, HbA1c, preoperative macular status, type of TRD), surgical characteristics (gauge, membrane peel, panretinal photocoagulation, type of tamponade), intervention details (timing and number of dexamethasone implants), and outcomes. The main anatomical outcome was an exploratory composite of study-reported postoperative PVR and/or retinal redetachment. Ocular-hypertension outcomes were extracted according to each study’s reported threshold and follow-up time point. Secondary outcomes included BCVA, CST, and postoperative complications. The unit of analysis was the eye. For each study, we recorded the number of patients and eyes, and whether bilateral eyes were included. Where bilateral eyes were included without adjustment for correlation between eyes from the same participant, this was considered a potential unit-of-analysis limitation. Discrepancies in extraction were resolved by consensus or by a third reviewer (A.S.H.). Study authors were contacted to clarify missing or unclear data. We also extracted reports of endophthalmitis, implant migration, cataract progression, silicone-oil emulsification, hypotony, and additional surgery.

Because individual fundus photographs, OCT volumes, and operative videos were unavailable, the reviewers did not independently grade preoperative or postoperative PVR. PVR status, redetachment, and complication outcomes were extracted according to definitions used in the original publications. This approach may introduce outcome misclassification and interpretation bias, particularly where baseline tractional membranes and postoperative PVR were not clearly separated.

### Quality assessment

The risk of bias for randomized controlled trials was assessed independently by two reviewers (T.M., E.F.) using the Cochrane Risk of Bias 2 tool (RoB 2) [9]. For non-randomized studies, the Risk of Bias in Non-randomized Studies of Interventions (ROBINS-I) tool was used [10]. Domains assessed included bias due to confounding, selection of participants, classification of interventions, deviations from intended interventions, missing data, measurement of outcomes, and selection of reported results. Disagreements were resolved through discussion with a third reviewer (A.J.Q.).

### Outcomes and statistical analysis

The main anatomical analysis was restricted to randomized controlled trials. The non-randomized study was analyzed separately as an exploratory sensitivity analysis because it had serious risk of bias due to confounding and participant selection. Because the studies used different anatomical definitions and follow-up times, the pooled anatomical result was described as an exploratory study-reported postoperative PVR/redetachment composite rather than a standardized PVR endpoint.

Ocular-hypertension outcomes were not assumed to have the same threshold or time point across studies. Early postoperative intraocular-pressure elevation, persistent ocular hypertension, medication-requiring ocular hypertension, and need for glaucoma surgery were recorded separately where reported.

Final 3-month logMAR BCVA values were pooled. For Zhang et al., which reported BCVA as median and interquartile range, the mean and standard deviation were estimated from the sample size, median, and interquartile range using the methods of Luo et al. and Wan et al. A sensitivity analysis excluding Zhang et al. was performed to assess the influence of these estimated values [11, 12].

All meta-analyses were performed using R software (version 4.4.3) with the meta package (version 8.2.1). For dichotomous outcomes, risk ratios (RRs) with 95% confidence intervals (CIs) were calculated using the inverse variance method under a random-effects model, with the between-study variance (τ²) estimated using the Restricted Maximum-Likelihood (REML) estimator. A continuity correction of 0.5 was applied to studies with zero-event cells where appropriate.

Given the small number of studies and sparse events, pooled estimates were interpreted cautiously. For dichotomous outcomes with zero events, sensitivity analyses were performed using alternative continuity corrections and/or risk difference models where feasible. Statistical significance was not interpreted as definitive evidence of efficacy when estimates were driven by few events or individual studies. A prespecified sensitivity analysis restricted to randomized trials was performed; the non‑randomized study was analyzed separately or included only in exploratory pooled analyses because of serious risk of bias.

Between-study heterogeneity was assessed using the I² statistic, with values interpreted as follows: <25% (low), 25–75% (moderate), and > 75% (substantial heterogeneity) [13]. Sensitivity analyses were planned to assess the robustness of the findings by (1) excluding studies judged to have a high overall risk of bias, and (2) re-running analyses using a fixed-effect model. The sensitivity analysis is provided in the Supplementary Materials (Supplementary Fig. 4).

Publication bias was not assessed via funnel plots or statistical tests due to the inclusion of fewer than ten studies per outcome, as such assessments are underpowered. The overall certainty (quality) of the evidence for each outcome was evaluated using the GRADE framework [14], considering risk of bias, inconsistency, indirectness, imprecision, and publication bias.

## Results

### Study selection and characteristics

The identification and selection of studies followed the PRISMA guidelines. A total of 132 records were identified through database searches (PubMed, Web of Science, Clinical Trials, Scopus). After removing 18 duplicates, 114 records were screened. Following primary screening, 109 records were excluded. The remaining five full-text articles were assessed for eligibility, and one was excluded in secondary screening (wrong population). Ultimately, four studies were included in the final meta-analysis.

The evidence base was small. Only four comparative studies met eligibility criteria, and only three were randomized trials. Sample sizes were modest, and the total number of dexamethasone‑treated eyes was limited. Therefore, all pooled estimates should be interpreted as exploratory.

The included analysis comprised four studies: three randomized trials (Zhang et al., 2025; Limon et al., 2022; Cao et al., 2025) and one non-randomized study (Iglicki et al., 2019). All three trials were judged to have some concerns overall in their risk of bias, primarily due to issues in selecting the reported result (Supplementary Fig. 2). The non-randomized study was assessed as having a serious overall risk of bias, largely stemming from critical concerns in confounding and participant selection (Supplementary Fig. 3). Across all studies, the domain of bias in selection of the reported result was most frequently identified as high risk.

A total of 305 eyes were analyzed. All studies enrolled patients with macula-off tractional retinal detachment (TRD) secondary to proliferative diabetic retinopathy (PDR). The intervention was pars plana vitrectomy (PPV) with silicone oil (SO) or heavy silicone oil (HSO) tamponade. The experimental group in all trials received an adjunctive intravitreal dexamethasone implant at the end of surgery, while the control groups received vitrectomy and tamponade alone. Baseline characteristics of the included studies are summarized in Table 1, and the corresponding primary and secondary outcome data are presented in Tables 2, 3 and 4.

**Table 1:** Characteristics of included studies

Study	Design	Eyes (*n*)	Population	Intervention	Control	Tamponade	Follow-up	Mean age (years)	TRD type
Cao 2025 [5]	RCT	34 (17 DEX / 17 control)	Adults ≥ 18 year, PDR stage 6 with RD	PPV + intraop. dexamethasone implant before SO	PPV + SO only	SO 1000 cSt	12 months	DEX 51.4 ± 10.7; Control 48.4 ± 11.6	TRD due to PDR
Zhang 2025 [4]	RCT	80 (40 DEX / 40 control)	Diabetic TRD requiring HSO tamponade	PPV + HSO + dexamethasone implant	PPV + HSO only	Heavy SO	3 months	DEX 57.8 ± 8.8; Control 56.2 ± 8.2	TRD due to PDR
Iglicki 2019 [2]	Non-RCT	148 (52 DEX / 96 control)	Macula-off, treatment-naïve diabetic TRD	PPV + PRP + SO + dexamethasone implant	PPV + PRP + SO	SO 1000 cSt	≥ 12 months (up to 24 months)	61.6 ± 15.8	TRD due to PDR
Limon 2022 [6]	RCT	43 (22 DEX / 21 control)	Type 2 DM, macula-off TRD + cataract	Phaco-PPV + SO + dexamethasone implant	Phaco-PPV + SO	SO 1000 cSt	6 months	DEX 56.7 ± 4.1; Control 59.8 ± 6.2	TRD due to PDR

DEX, dexamethasone implant; HSO, heavy silicone oil; PDR, proliferative diabetic retinopathy; PPV, pars plana vitrectomy; PRP, panretinal photocoagulation; RCT, randomized controlled trial; RD, retinal detachment; SO, silicone oil; TRD, tractional retinal detachment

All studies enrolled macula-off TRD due to PDR. A single intraoperative dexamethasone implant was used in all studies unless noted otherwise. Iglicki et al. was non-randomized; group sizes reflect the original treatment allocation (52 dexamethasone implant, 96 control). The unit of analysis was the eye. Cao et al. included 34 eyes from 30 participants; this may introduce correlation between eyes from the same participant. Bilateral-eye inclusion was not clearly reported for all remaining studies

**Table 2:** Study-specific anatomical and intraocular-pressure outcomes

Study	Study-reported postoperative proliferative/redetachment outcome	Study-reported intraocular-pressure outcome
Cao 2025 [5]	NR for the exploratory PVR/redetachment composite.	Reported increased IOP: 7/17 vs. 6/17; exact threshold and assessment time point were not harmonized.
Zhang 2025 [4]	Study-reported postoperative proliferative/redetachment outcome, 3 months: 4/40 vs. 18/40.	No dichotomous IOP event data were included in the review analysis.
Limon 2022 [6]	Study-reported PVR/redetachment outcome, 6 months: 0/22 vs. 6/21.	Study-reported IOP outcome: 2/22 vs. 3/21; definition and time point were study-specific.
Iglicki 2019 [2]	Study-reported PVR/redetachment outcome, study-specific follow-up: 2/52 vs. 46/71. Exploratory sensitivity analysis only.	Study-reported IOP outcome: 2/52 vs. 4/96; definition and time point were study-specific.

DEX, dexamethasone implant; IOP, intraocular pressure; NR, not reported; PVR, proliferative vitreoretinopathy

Definitions and postoperative assessment time points differed across studies. Intraocular-pressure outcomes were therefore not pooled. Iglicki et al. was included only in the exploratory anatomical sensitivity analysis because of serious risk of bias

Numerators and denominators are shown as dexamethasone implant versus control, as reported by the original studies

**Table 3:** Secondary outcome data: final BCVA at 3 months and central thickness at 6 months

Study	Final 3-month BCVA (logMAR), mean ± SD		Central thickness at 6 months (µm), mean ± SD	
	DEX	Control	DEX	Control
Cao 2025 [5]	0.80 ± 0.15	1.20 ± 0.05	223 ± 118.9	275.5 ± 131.9
Zhang 2025 [4]	0.51 ± 0.40	0.79 ± 0.65	NR	NR
Limon 2022 [6]	0.74 ± 0.21	0.94 ± 0.19	214 ± 98.6	293 ± 86.7
Iglicki 2019 [2]	NR	NR	282 ± 25	441 ± 141

BCVA, best-corrected visual acuity; DEX, dexamethasone implant; NR, not reported; SD, standard deviation

Zhang et al. reported median and interquartile-range BCVA values; mean and SD were estimated using the methods of Luo et al. and Wan et al. [11, 12]

All-study BCVA pooled MD: −0.30 logMAR (95% CI − 0.58 to − 0.03; I² = 74.9%). Sensitivity analysis excluding Zhang et al.: MD − 0.31 logMAR (95% CI − 1.57 to 0.96; I² = 87.0%). CST pooled MD: −104.13 μm (95% CI − 243.82 to 35.56; I² = 77.8%)

**Table 4:** GRADE summary of findings

Outcome	Studies / eyes	Effect estimate	Certainty	Reasons for downgrading
Exploratory postoperative proliferative/redetachment composite (RCTs only)	2 / 123	RR 0.20 (95% CI 0.00 to 16.06). Crude event proportions: 4/62 vs. 24/61.	Very low	Risk of bias; indirectness due to non-equivalent endpoint definitions and time points; very serious imprecision from sparse events and an extremely wide CI.
Final BCVA at 3 months	3 / 157	MD − 0.30 logMAR (95% CI − 0.58 to − 0.03); sensitivity excluding Zhang: MD − 0.31 (95% CI − 1.57 to 0.96).	Very low	Risk of bias; serious inconsistency (I² = 74.9%); imprecision; conversion of median/IQR data for Zhang et al.
Central thickness at 6 months	3 / 192	MD − 104.13 μm (95% CI − 243.82 to 35.56).	Very low	Risk of bias; serious inconsistency (I² = 77.8%); imprecision.
Study-reported intraocular-pressure outcomes	3 / 225	Not pooled because thresholds, time points, and treatment reporting differed.	Very low	Risk of bias; indirectness and inconsistency due to heterogeneous outcome definitions; imprecision; no pooled relative effect.

### Primary outcomes

#### Exploratory postoperative proliferative/redetachment outcome

**Fig. 1:**
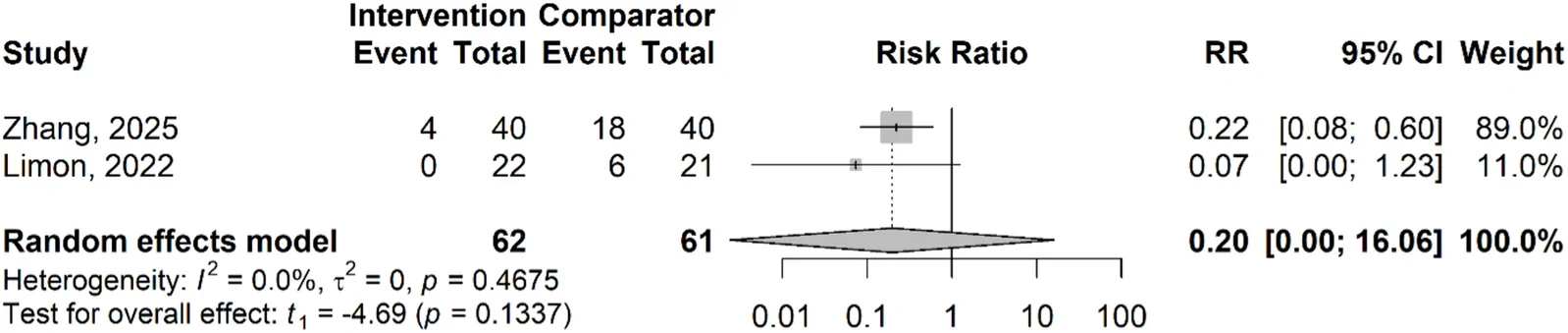
Exploratory study-reported postoperative proliferative/redetachment composite: randomized trials only. Forest plot of two randomized trials. The random-effects risk ratio was 0.20 (95% CI 0.00 to 16.06; I² = 0%). The estimate was highly imprecise and inconclusive

Two randomized trials comprising 123 eyes contributed to the main exploratory study-reported postoperative proliferative/redetachment composite. The random-effects pooled risk ratio was 0.20 (95% CI 0.00 to 16.06; I² = 0%; Fig. 1). Although the point estimate favored dexamethasone implant, the confidence interval was extremely wide and crossed the null value; therefore, the randomized-trial evidence was inconclusive. The outcome definitions and postoperative assessment time points differed between studies and were not interpreted as a uniformly graded PVR endpoint.

In an exploratory sensitivity analysis including the non-randomized Iglicki study, the pooled risk ratio was 0.12 (95% CI 0.02 to 0.83; I² = 21%). Because Iglicki et al. had serious risk of bias due to confounding and participant selection, this result was not used as the main estimate of effect.

#### Study-reported intraocular-pressure elevation

Three studies reported postoperative intraocular-pressure outcomes. However, the definitions of elevated intraocular pressure, assessment time points, and reporting of treatment requirements differed between studies. Therefore, intraocular-pressure outcomes were summarized descriptively rather than pooled. Study-specific definitions, timing, and management are presented in Table 2 and Supplementary Table 2.

### Secondary outcomes

#### Final BCVA at 3 months

**Fig. 2:**
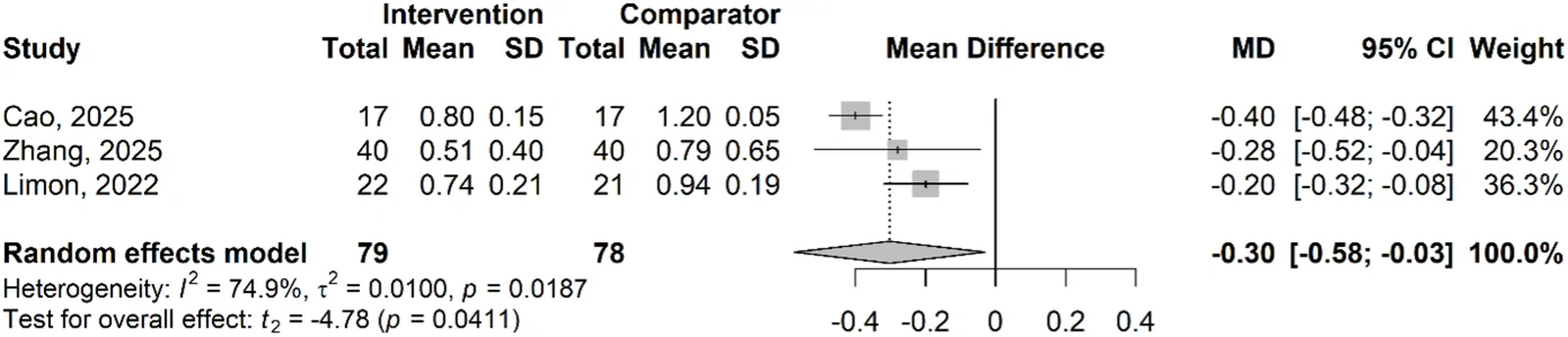
Final best-corrected visual acuity at 3 months. Forest plot of final 3-month logMAR BCVA values. The all-study random-effects mean difference was − 0.30 logMAR (95% CI − 0.58 to − 0.03; I² = 74.9%). Zhang et al. required median/IQR-to-mean/SD conversion; the sensitivity analysis excluding Zhang et al. was inconclusive

Final 3-month logMAR BCVA was analysed as an exploratory outcome. In the analysis including all three studies, the pooled mean difference was − 0.30 logMAR (95% CI − 0.58 to − 0.03; I² = 74.9%; Fig. 2). Zhang et al. reported median and interquartile-range values; estimated mean and standard deviation values were derived using the methods of Luo et al. and Wan et al. In the sensitivity analysis excluding Zhang et al., the pooled mean difference was − 0.31 logMAR (95% CI − 1.57 to 0.96; I² = 87.0%). The wide confidence interval and substantial heterogeneity mean that the BCVA finding remains uncertain.

#### Exploratory 6-month central thickness outcome

**Fig. 3:**
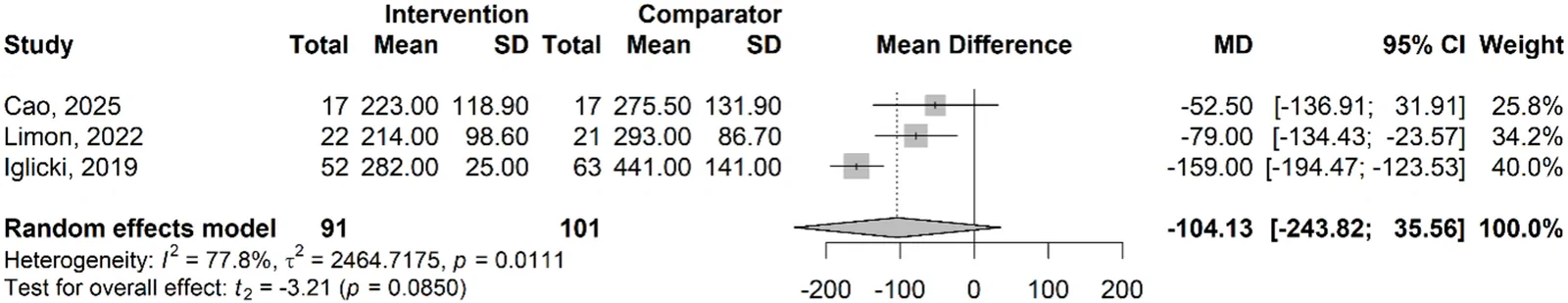
Exploratory central thickness outcome at 6 months. Forest plot comparing central thickness at 6 months. The random-effects mean difference was − 104.13 μm (95% CI − 243.82 to 35.56; I² = 77.8%), with high heterogeneity. The estimate was exploratory and inconclusive

Central subfield thickness (CST) at 6 months was analyzed in three studies comprising 192 eyes. The pooled mean difference favored dexamethasone but did not reach statistical significance, and the confidence interval was very wide (MD = -104.13 μm; 95% CI -243.82 to 35.56; *p* = 0.08), with high heterogeneity (I² = 77.8%, *p* = 0.01). Sensitivity analysis revealed that omission of Iglicki (2019) markedly reduced heterogeneity (I² = 0%), indicating that this study contributed disproportionately to the variability. Substantial heterogeneity limits interpretation, likely reflecting differences in baseline macular status, surgical complexity, tamponade type, OCT timing, and diabetic macular edema activity. The imprecise estimate precludes any firm conclusion about a treatment effect on CST (Fig. 3).

### Statistical limitations

This meta-analysis had several statistical limitations. The number of trials and total sample size were modest, especially the three prospective RCTs, leading to wide confidence intervals and limited power to detect modest differences between the groups. Several outcomes showed substantial between-study heterogeneity, and the analyses were underpowered to detect modest effects. In addition, extreme event rates (near-total prevention of PVR in the dexamethasone group) may inflate risk ratio estimates and contribute to imprecision. All pooled estimates should be viewed as exploratory rather than definitive.

## Discussion

This systematic review identified four comparative studies evaluating adjunctive dexamethasone implant during PPV with silicone oil or heavy silicone oil for diabetic TRD. In the main randomized-trial analysis, the exploratory study-reported postoperative proliferative/redetachment composite was highly imprecise and inconclusive. The confidence interval was extremely wide, and the available studies used heterogeneous anatomical definitions and follow-up time points.

An exploratory sensitivity analysis including the non-randomized Iglicki study favored dexamethasone implant; however, this study had serious risk of bias and was not used as the primary estimate. Therefore, the available evidence should be considered hypothesis-generating rather than evidence of a clinically significant reduction in PVR.

### Challenges in defining PVR after diabetic TRD surgery

In diabetic TRD, postoperative retinal distortion may reflect residual traction, incomplete membrane removal, recurrent fibrovascular proliferation, iatrogenic breaks, or true postoperative PVR. Grade B PVR features are particularly difficult to interpret because retinal surface wrinkling, stiffness, and vascular tortuosity may be present before surgery. For this reason, redetachment attributable to grade C or worse PVR, or need for reoperation due to PVR, may be a more clinically meaningful endpoint than study‑reported PVR grade B/C.

Final 3-month BCVA findings were uncertain. Although the all-study analysis favored dexamethasone implant, substantial heterogeneity was present and Zhang et al. required conversion of median and interquartile-range data to estimated mean and standard deviation. When Zhang et al. was excluded, the confidence interval crossed the null value and heterogeneity remained substantial. Central thickness findings were also uncertain because of high heterogeneity and imprecision. Intraocular-pressure outcomes were not pooled because definitions and postoperative assessment time points differed between studies.

To the best of our knowledge, this is the first quantitative synthesis of research assessing the use of adjuvant dexamethasone implants in patients having silicone oil vitrectomy for macula‑off diabetic TRD. Our results are consistent with the findings of the included studies [2, 4–6], which reported that supplementary dexamethasone decreased the incidence or severity of PVR. The direction of some study-level findings favored dexamethasone implant; however, the evidence remains insufficient to establish a clinically significant advantage. Proteomic findings demonstrating elevated levels of pro‑inflammatory cytokines (IL‑6, IL‑8), complement proteins, and matrix metalloproteinases in the vitreous of eyes with retinal detachment and PVR provide a rationale for corticosteroid use in this situation [15].

Previous trials evaluating corticosteroids for PVR have yielded mixed results. Koerner et al. [16] found that systemic corticosteroids reduced the risk of epiretinal membrane formation after retinal detachment surgery but did not prevent PVR progression. More relevantly, Banerjee et al. [17] reported no benefit of slow-release dexamethasone for treating established PVR. In contrast, the present meta-analysis examines dexamethasone as a prophylactic strategy initiated at the time of surgical repair in a high-risk diabetic population. The potent and sustained anti-inflammatory effect of the dexamethasone implant, combined with its delivery at the moment of maximal surgical insult, may explain the more favorable outcomes observed in this specific context.

The pharmacokinetic behavior of dexamethasone implants in vitrectomized, silicone oil-filled eyes is a crucial consideration for therapeutic use. Preclinical research has shown that the dexamethasone implant’s pharmacokinetic profile is comparable in vitrectomized and non-vitrectomized eyes, with sustained drug levels maintained for a minimum of 31 days [18]. This implies that the delivery qualities of the implant are not compromised by vitrectomy. However, in vitro data suggest that the viscosity of silicone oil may change the anti-inflammatory effectiveness and dexamethasone release kinetics, with denser oils possibly reducing the drug’s action even at detectable doses [19]. These results emphasize the need for more research on the best times to implant and what kind of oil to use. Although pooled estimates from randomized trials have not reached statistical significance (RR = 0.64; 95% CI: 0.30 to 1.38) [20], emerging evidence for alternative adjunctive agents, such as methotrexate, has shown promise in PVR prevention, highlighting the need for bigger, conclusive research.

The certainty of evidence for the exploratory postoperative proliferative/redetachment composite was very low because of risk of bias, sparse events, very serious imprecision, and indirectness related to inconsistent outcome definitions and follow-up time points. The estimate was not sufficiently robust to support a high-confidence conclusion.

### Strengths and limitations

The principal strength of this review is that it summarizes the entire currently available comparative evidence on a focused surgical question. However, the evidence base remains limited rather than definitive. Key limitations include the small number of studies (only four, of which three were randomized), the modest total dexamethasone-treated sample, and the serious risk of bias in the non-randomized study. PVR was not independently graded from standardized images, and published descriptions may not distinguish baseline traction from new postoperative PVR. Furthermore, grade B PVR is not a reliable endpoint in diabetic TRD, and baseline disease severity, macular status, HbA1c, duration of diabetes, tamponade type, and surgical complexity may confound outcomes. Follow-up was mostly short-term, and publication bias could not be assessed (funnel plots and statistical tests were precluded by the small number of studies).

Wide confidence intervals and low ability to identify moderate treatment effects, especially for secondary outcomes, were caused by the small aggregate sample size. The majority of trials had a 6-month follow-up period, which left long-term anatomic and functional results unknown. The significant variation in secondary outcomes like BCVA and CST was probably caused by differences in tamponade type, the precise time of dexamethasone implant insertion, and PVR grading techniques. Furthermore, subgroup analyses that would have shown impact modifiers like glycemic control (HbA1c), the length of diabetes, or the degree of baseline macular detachment were not possible due to the lack of individual patient data. The non-randomized study by Iglicki (2019) was assessed as having a serious overall risk of bias, which might affect the pooled results.

### Clinical implications

At present, the evidence is insufficient to recommend routine dexamethasone implantation for all diabetic TRD cases undergoing silicone oil tamponade. The available data may support selective consideration in highly inflammatory or high-risk cases, but this decision should remain individualized until adequately powered randomized trials confirm benefit using standardized anatomical endpoints.

## Conclusion

In this systematic review and exploratory meta-analysis, the randomized-trial evidence for adjunctive dexamethasone implant during PPV with silicone oil or heavy silicone oil for diabetic TRD was inconclusive. The exploratory postoperative proliferative/redetachment composite was highly imprecise, and studies used heterogeneous outcome definitions and follow-up time points. An exploratory analysis including a non-randomized study favored dexamethasone implant but was affected by serious risk of bias. BCVA and central-thickness outcomes remained uncertain, and intraocular-pressure outcomes could not be pooled. Current evidence does not support routine use; dexamethasone implant should remain a selective, individualized consideration pending adequately powered randomized trials with standardized anatomical outcomes.

## Supplementary Information

Below is the link to the electronic supplementary material.

Supplementary Material 1

## Data Availability

The data supporting the findings of the manuscript will be made available upon request to the corresponding author.
