# Supplementary material for: Adjunctive dexamethasone implant during vitrectomy with silicone oil for diabetic tractional retinal detachment: a systematic review and exploratory meta-analysis
Source: Int J Retina Vitreous. 2026 Jul 21;12:139. doi: 10.1186/s40942-026-00903-5 (PMC13679000; doi:10.1186/s40942-026-00903-5)
Supplement: Supplementary file 1 [file 40942_2026_903_MOESM1_ESM.docx]

**Supplemental Material**

**Supplementary Figure 1. PRISMA flow diagram of study selection.**

**Supplementary Figure 2. Risk of bias in randomised trials (RoB 2).**

**Supplementary Figure 3. Risk of bias in the non‑randomised study (ROBINS‑I).**

**Supplementary Figure 4. Sensitivity analyses.**

**Supplementary Table 1. Database search strategies used in the review.**

**Supplementary Table 2. Study-specific intraocular-pressure outcomes.**

**Supplementary Figure 1. PRISMA flow diagram of study selection.**

Flow diagram showing the number of records identified, screened, assessed for eligibility, and included. Of 132 database results, four studies (three RCTs, one non‑randomised) met the inclusion criteria.

**Supplementary Figure 2. Risk of bias in randomised trials (RoB 2).**

Domain‑level and overall risk‑of‑bias judgements for the three included RCTs, assessed with the Cochrane RoB 2 tool. All three trials were rated as having “some concerns” overall.

**Supplementary Figure 3. Risk of bias in the non‑randomised study (ROBINS‑I).**

Domain‑level and overall risk‑of‑bias judgement for the single non‑randomised study (Iglicki 2019), assessed with ROBINS‑I. The overall rating was “serious” due to confounding and participant selection concerns.

**Supplementary Figure 4. Sensitivity analyses.**

(a) Exploratory anatomical sensitivity analysis including Iglicki et al.

(b) Final 3-month BCVA sensitivity analysis excluding Zhang et al.

(c) Central-thickness sensitivity analysis excluding Iglicki et al.

Sensitivity analyses for (a) the exploratory anatomical outcome including the non-randomized Iglicki study, (b) final 3-month BCVA excluding Zhang et al., and (c) central thickness excluding Iglicki et al. Panel A is exploratory because the Iglicki study had serious risk of bias. Panel B assesses the influence of the median/IQR-to-mean/SD conversion used for Zhang et al. Panel C shows the influence of the non-randomized study on central-thickness heterogeneity.

**Supplementary Table 1. Database search strategies used in the review.**

| **Databases searched** | **Search string** | **Articles retrieved** | **Date last searched** |
| --- | --- | --- | --- |
| PubMed | ((“diabetic tractional retinal detachment”[tiab]  OR “tractional retinal detachment”[tiab]  OR “TRD”[tiab]))  AND  ((“pars plana vitrectomy”[tiab] OR PPV[tiab])  OR silicone[tiab] OR “silicone oil”[tiab]  OR “heavy silicone oil”[tiab])  AND  (“dexamethasone implant”[tiab] OR Ozurdex[tiab]  OR dexamethasone[tiab]) | 96 | 29^th^ December 2025 |
| Web of Science | TS = ("tractional retinal detachment" OR "diabetic TRD")  AND TS = ("pars plana vitrectomy" OR PPV OR "silicone oil" OR "heavy silicone oil")  AND TS = ("dexamethasone implant" OR Ozurdex OR dexamethasone) | 2 | 29^th^ December 2025 |
| Clinicaltrials.gov | Search:   - “dexamethasone implant” + “vitrectomy” - “tractional retinal detachment” - “diabetic retinopathy” + “silicone oil”   Extract:   - completed trials - unpublished datasets - protocols for bias assessment | 30 | 29^th^ December 2025 |
| Scopus | ("diabetic tractional retinal detachment" OR "tractional retinal detachment")  AND ("pars plana vitrectomy" OR PPV OR "silicone oil" OR "heavy silicone")  AND ("dexamethasone implant" OR Ozurdex OR dexamethasone) | 4 | 29^th^ December 2025 |

**Supplementary Table 2. Study-specific intraocular-pressure outcomes.**

| **Study** | **Study-reported intraocular-pressure outcome** |
| --- | --- |
| Cao 2025 [5] | Reported increased IOP: 7/17 vs 6/17; exact threshold and assessment time point were not harmonized. |
| Zhang 2025 [4] | No dichotomous IOP event data were included in the review analysis. |
| Limon 2022 [6] | Study-reported IOP outcome: 2/22 vs 3/21; definition and time point were study-specific. |
| Iglicki 2019 [2] | Study-reported IOP outcome: 2/52 vs 4/96; definition and time point were study-specific. |

IOP, intraocular pressure. Other postoperative complications, including endophthalmitis, implant migration, cataract progression, silicone-oil emulsification, hypotony, and additional surgery, were not consistently reported in the extracted study data.
